# Defective in utilizing glutathione 3, *DUG3,* is required for conidiation and host infection in the rice blast fungus *Magnaporthe oryzae*

**DOI:** 10.17912/micropub.biology.000550

**Published:** 2022-04-15

**Authors:** Md. Hashim Reza, Kaustuv Sanyal

**Affiliations:** 1 Molecular Biology and Genetics Unit, Jawaharlal Nehru Centre for Advanced Scientific Research, Jakkur, Bengaluru 560 064, India; 2 Bharat Chattoo Genome Research Centre, Department of Microbiology & Biotechnology Centre, Faculty of Science, The Maharaja Sayajirao University of Baroda, Vadodara 390 002, Gujarat, India

## Abstract

Glutathione, an important redox buffer of the cell, also functions as a source of sulphur and nitrogen under starvation conditions. The metabolism and maintenance of glutathione homeostasis are vital for the appropriate functioning of the cell. In addition to the γ-glutamyl transpeptidase, the fungus-specific alternative pathway involving *DUG1, DUG2* and *DUG3* genes also mediate glutathione degradation. Here, we studied the functional significance of *DUG3* in the vegetative growth and infection cycle of the cereal blast fungus *Magnaporthe oryzae* . Cells lacking the *DUG3* gene displayed reduced conidiation, delayed appressorium formation, and a decrease in the severity of host infection. Further, we show that the γ-glutamyl transpeptidase inhibitor severely compromises the vegetative growth of the *M. oryzae* cells lacking the *DUG3* gene. Taken together, our results suggest a significant role of glutathione metabolism in the growth and virulence of *M. oryzae* .

**Figure 1. M. oryzae cells lacking the DUG3 gene are defective in melanization, conidiation, appressorium formation, and host infection:**
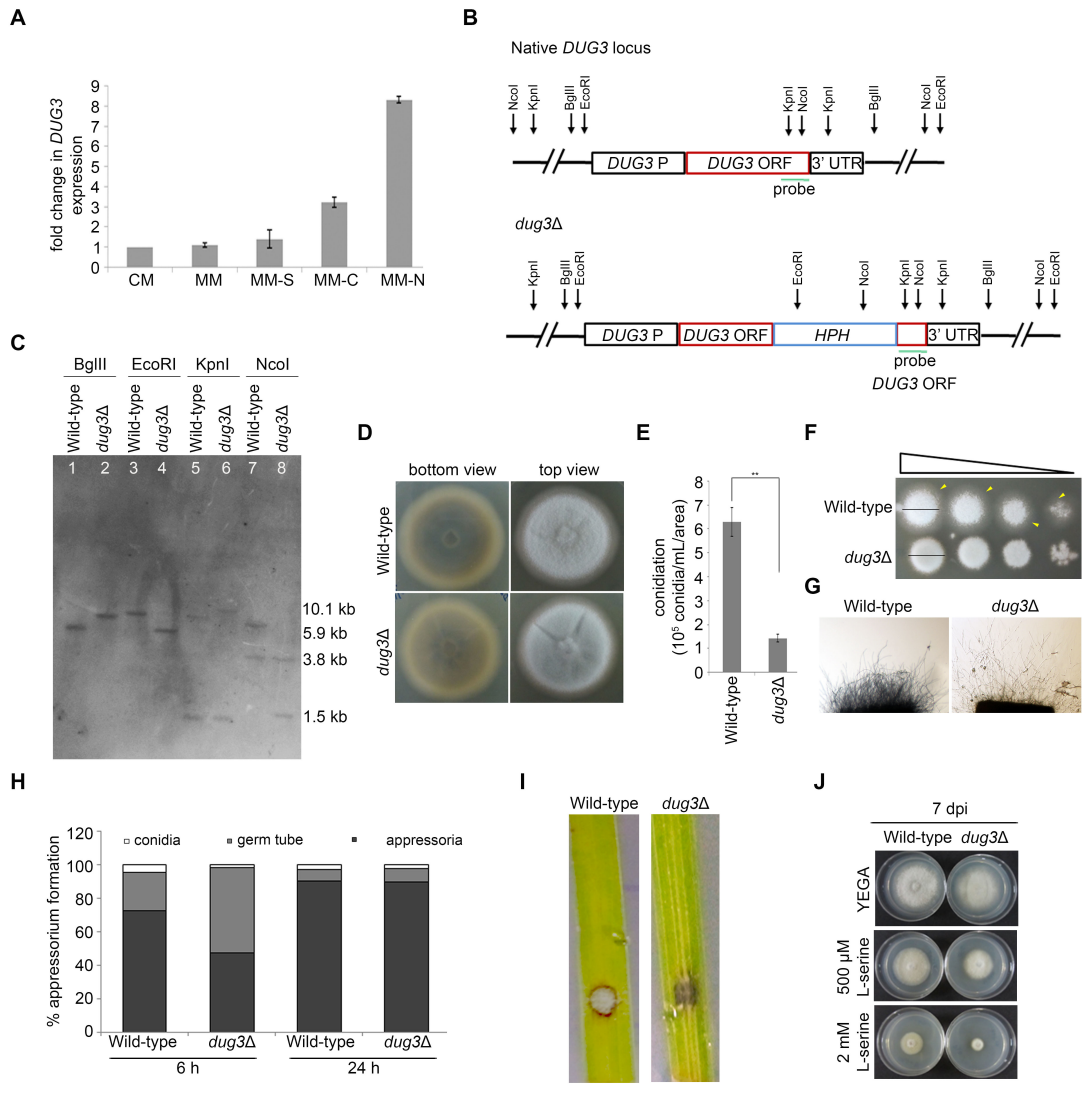
(A) Transcript levels of *DUG3* were estimated by qPCR in wild-type *M. oryzae* grown in complete medium (CM), minimal medium (MM), minimal medium without a sulphur (MM-S), carbon (MM-C) or nitrogen (MM-N) source. Error bars represent SD. (B) Schematic of the native *DUG3* gene locus and the corresponding deletion cassette used to replace the *DUG3* gene to generate a *dug3* null mutant strain. The restriction enzymes and the probe DNA region (green line) used in Southern hybridization are marked. (C) Southern hybridization to validate the targeted replacement of the *DUG3* gene with the deletion cassette in the genome of *M. oryzae* . The wild-type and *dug3* ∆ genomic DNA was digested with BglII, EcoRI, KpnI, and NcoI and probed with the fragment as shown in (B). The expected fragment sizes when digested with BglII (wild-type, 5.8 kb; *dug3* ∆, 7.7 kb), EcoRI (wild-type, 8.6 kb; *dug3* ∆, 5.9 kb), KpnI (wild-type, 1.3 kb+8.2 kb; *dug3* ∆, 1.3+10.1 kb), and NcoI (wild-type, 3.8+7.1 kb; *dug3* ∆, 3.8+1.5 kb) are marked on the right. (D) Vegetative growth of wild-type and *dug3* ∆ strains showing melanization (bottom view) and hyphal growth (top view) grown on OMA at 8 days post-inoculation (dpi). (E) Bar graph displaying mean ± SEM conidiation frequency of wild-type and *dug3* ∆ strains. (F) Spores of wild-type and *dug3* ∆ strains were 10-fold serially diluted and spotted on YEGA. Vegetative growth was scored at 5 dpi. The black lines and yellow arrowheads mark the diameter and feeding hyphal growth respectively of the fungal colony. (G) Microscopic examination of hyphal growth of wild-type and *dug3* ∆ strains were assessed after 2 dpi grown on 0.8% agarose. (H) Bar graph displaying appressorium frequency of wild-type and *dug3* ∆ strains at 6 and 24 hpi on a hydrophobic surface. (I) Detached leaf assay in a susceptible variety HR12 cultivar was carried out with spores (10 ^4^ mL ^-1^ ) of wild-type and *dug3* ∆ strains. Disease symptoms (lesions) were assessed at 4 dpi. (J) Vegetative growth of wild-type and *dug3* ∆ strains grown on YEGA or in presence of borate buffer with L-serine (500 µM and 2 mM), a γ-glutamyl transpeptidase inhibitor and photographed after 7 dpi.

## Description

*M. oryzae,* the causative agent of the cereal blast disease, has emerged as a model pathogen to study host-pathogen interactions and tops the list of plant pathogens due to its economic significance (Dean et al., 2012). It is a major concern for agriculture-dependent economies and world food security. *M. oryzae* under nutrient deprivation conditions germinates and differentiates into appressorium, the infection structure, and penetrates the host tissue forming primary invasive hyphae. During early invasive growth, *M. oryzae* derives energy via vacuolar turnover of its organelles and macromolecules and therefore vacuolar dynamics and function are vital for its successful establishment within the host (Reza et al., 2021). Additionally, the pathogen also encounters a defence response from the host which is established by a rapid burst of reactive oxygen species (ROS) and cell death at the site of invasion (Apostol et al., 1989). Glutathione not only functions as a primary redox buffer neutralizing the oxidative stress response but also plays a role in the stabilization of yeast vacuolar function (Sharma et al., 2003). The higher intracellular levels of glutathione (0.1-10 mM) are maintained by the unusual γ-glutamyl bond, making it resistant to peptidases in the cell (Hwang et al., 1992). *Saccharomyces cerevisiae* has evolved mechanisms to metabolize glutathione during sulphur and nitrogen starvation conditions, either by upregulating γ-glutamyl transpeptidase or employing Dug1-3 (having amino transpeptidase activity), enzymes that cleave glutathione, thereby releasing glycine, glutamate, and cysteine which enables the cell to utilize these amino acids as nitrogen and sulphur source (Ganguli et al., 2007, Kumar et al., 2003). Glutathione is mobilized towards vacuoles under nitrogen starvation ensuring cellular maintenance in nutritional stress. With the alternative pathway of glutathione degradation being specific to fungi (Kaur et al., 2012), and *M. oryzae* experiencing starvation conditions during pathogenic development, this study improves our understanding of the role of *DUG3* and glutathione metabolism during the infection cycle of the cereal blast fungus.

The *M. oryzae* genome encodes for a single *DUG3* (Mgg_11745) gene which is 1684 bp long with three introns and is predicted to express a 469-amino acid long protein. *M. oryzae* Dug3 protein shows 47% identity with its *S. cerevisiae* homolog and has the conserved glutamine amidotransferase type 2 domain spanning from 38-306 amino acids as predicted by Pfam database. To address whether *DUG3* plays any role during starvation conditions in *M. oryzae* and is expressed under these conditions, we studied the transcript level of *DUG3* by qPCR. Remarkably, *DUG3* was expressed 3-fold and 8-fold under carbon and nitrogen starvation respectively when compared to a nutrient-rich complete medium ( **Figure 1A** ), suggesting a likely role of *DUG3* under carbon and nitrogen starvation conditions in utilizing glutathione as a nutrient source. To understand the role of *DUG3* in the growth and development of *M. oryzae* , the gene was disrupted at the native locus by *Agrobacterium tumefaciens* -mediated transformation and the transformants were validated by Southern blot analysis ( **Figures 1B and C** ). *DUG3* is a non-essential gene in *M. oryzae* and loss of its function did not show any evident defects in its vegetative growth, with the colony diameter of the *dug3* ∆ transformant being comparable to that of the wild-type when fungal mycelia were used as an inoculum ( **Figure 1D** ). However, the *dug3* ∆ cells displayed less melanization as compared to the wild-type ( **Figure 1D** ). To assess the asexual development in the *dug3* ∆ transformant, we studied conidiation. The role of *DUG3* in conidiation was established with the *dug3* ∆ transformant displaying a ~4.4-fold decrease in conidiation ( **Figure 1E** ). When these conidia were kept for germination, the *dug3* ∆ transformant displayed a reduced feeding hyphal growth ( **Figure 1F** ) and a reduced aerial hyphal growth ( **Figure 1G** ), suggesting the role of *DUG3* during conidial germination and hyphal growth. To assess the role of *DUG3* in the pathogenesis of *M. oryzae* , we next studied the appressorial development in the *dug3* ∆ transformant by an *in vitro* assay using an inductive hydrophobic glass surface. The *dug3* ∆ transformant displayed a ~1.5-fold decrease in appressorium formation at 6 hpi ( **Figure 1H** ). However, by 24 hpi the appressorium formation was comparable between the *dug3* ∆ transformant and wild-type ( **Figure 1H** ), suggesting a delay in appressorium formation with loss of *DUG3* . Next, we assayed the virulence of the wild-type and the *dug3* ∆ transformant on rice leaf explants. The wild-type formed a typical disease lesion (marked by a greyish centre and brown margins), while the *dug3* ∆ transformant failed to form a typical lesion ( **Figure 1I** ), indicating the importance of *DUG3* during the infection cycle of *M. oryzae* .

Finally, to study the effect of glutathione degradation inhibition on vegetative growth, the wild-type and *dug3* ∆ transformants were grown in presence of γ-glutamyl transpeptidase inhibitor, L-serine, which inhibits the classical γ-glutamyl pathway of glutathione degradation (Tate and Meister, 1978). When compared to the control (YEGA), even the wild-type cells showed growth inhibition with increasing concentration of the inhibitor ( **Figure 1J** ). However, the *dug3* ∆ transformant, having the alternate pathway of glutathione degradation perturbed, showed enhanced growth defect in presence of the inhibitor ( **Figure 1J** ). Taken together, these findings suggest that glutathione degradation, either by the γ-glutamyl pathway or an alternate pathway involving *DUG* genes, contributes to the growth and pathogenic development of *M. oryzae* . It remains to be addressed whether *DUG3* plays a role in host penetration, primary invasive hyphae formation, and the secondary invasive hyphae formation stage of pathogenic development. It will be also worth addressing the change in the intracellular levels of glutathione during the *M. oryzae* life cycle.

## Methods

**Fungal strains, culture conditions, and transformation:** *M. oryzae* B157 strain (MTCC accession number 12236), belonging to the international race IC9 (Kachroo et al., 1994), was grown and maintained on oat meal agar (OMA) or yeast extract dextrose agar (YEGA). Liquid complete medium (CM) was used for growing fungal biomass for DNA or RNA isolation. Vegetative growth was measured in terms of colony diameter on OMA or YEGA. Conidia were harvested after growing the cultures for 9 days on OMA and total conidia were harvested as described earlier (Reza et al., 2016). Gene deletion constructs were transferred into *M. oryzae* by *Agrobacterium tumefaciens* -mediated transformation (Mullins et al., 2001).

For the spore germination and growth assay on YEGA, 10-fold serially diluted spores of wild-type and *dug3* ∆ strains were spotted and colony morphology was assessed 5 dpi. For the growth assay in presence of γ-glutamyl transpeptidase inhibitor, fungal mycelia of wild-type and *dug3* ∆ strains were inoculated on YEGA and YEGA supplemented with 2.5 mM borate buffer having either 500 µM or 2 mM L-serine (Tate and Meister, 1978). The growth was assessed after 7 dpi.

***DUG3* deletion cassette generation:** The pGKO2- *DUG3* - *HPH* construct was generated for carrying out targeted disruption of *DUG3* in wild-type B157 strain. Full-length *DUG3* ORF (~ 1684 bp) was cloned at XhoI and XbaI sites in pGKO2 vector having T-DNA borders to give pGKO2- *DUG3* . The *HPH* cassette used for disrupting *DUG3* was amplified from pSilent and cloned at HindIII site in between the *DUG3* gene to give pGKO2- *DUG3* - *HPH* . The *A. tumefaciens* strain LBA4404/pSB1 was first transformed with pGKO2- *DUG3* - *HPH* via triparental mating using helper plasmid pRK2013. The transformed *Agrobacterium* was used to carry out *A. tumefaciens* -mediated transformation of *M. oryzae* . The transformants were selected on YEGA with 200 µg mL ^-1^ Hygromycin. The transformants were screened by locus-specific PCR and further validated by Southern blot hybridization.

**Appressorial assay:** The appressorial assay was carried out by inoculating an equal number of conidia of wild-type and *dug3* ∆ strains on a hydrophobic surface, Gelbond film (Amersham Pharmacia Biotech AB, Uppsala, Sweden) and incubated in a moist chamber for 6 h and 24 h. A minimum of 100 conidia were counted for their ability to form appressoria and germ tubes.

**qPCR for *DUG3* gene expression:** Wild-type fungal mycelia were grown in complete medium (CM) for 48 h at 28°C and 200 rpm. The fungal biomass was washed twice with autoclaved water and the biomass was resuspended in CM, minimal medium (MM), minimal medium without sulphur (MM-S)/ carbon (MM-C)/ nitrogen (MM-N) source for 24 h. The biomass was harvested and snap-frozen in liquid nitrogen. Total RNA was isolated using TRIzol (Invitrogen Life Technologies, California, USA). Two µg of RNA was used to reverse transcribe first-strand cDNA using oligo dT primer and M-MuLV reverse transcriptase (NEB, Massachusetts, USA). qPCR was carried out by monitoring the increase in fluorescence of SYBR green dye on a Light Cycler system (Roche Applied System, Mannheim, Germany). To compare the relative abundance of *DUG3* , the average threshold cycle (Ct) was normalized to that of tubulin for each of the treated samples as 2 ^-∆Ct^ , where ∆Ct = (Ct *_DUG3_* -Ct _tubulin_ ) and fold change was calculated by 2 ^-∆∆Ct^ , where ∆∆Ct = (Ct *_DUG3_* -Ct _tubulin_ ) _test condition_ - (Ct *_DUG3_* -Ct _tubulin_ ) _control_ .

**Nucleic acid manipulation and Southern blotting:** Genomic DNA was extracted as described (Dellaporta et al., 1983). Southern blot analysis was performed as previously described (Sambrook and Russell, 2001). Ten µg genomic DNA of wild-type and *dug3* ∆ strains were digested with BglII, EcoRI, KpnI, and NcoI and the blot was probed with ~ 600 bp fragment as shown in Figure 1B. The probes were labelled and hybridizing bands were detected using Gene Images AlkPhos Direct Labeling and Detection system as per the manufacturer’s instructions (Amersham, Buckinghamshire, England).

**Aerial hyphal growth:** Aerial hyphal growth was assessed by growing both wild-type and *dug3* ∆ strains on a glass slide containing 0.8% agarose for 2 days. Inoculated cultures were maintained in a humid chamber at 28°C (Reza et al., 2016).

**Infection assay:** Detached leaf assay was carried out in a susceptible variety HR12 cultivar by droplet inoculation of conidial suspension. Leaves of the 21-day old plants were used for inoculating 10 ^4^ mL ^-1^ spores of wild-type and *dug3* ∆ strains. The leaves were placed on water agar with kinetin (2mg mL ^-1^ ). The inoculated leaves were maintained in a humid chamber at 26°C and disease symptoms (lesions) were assessed at 4 dpi.
